# Free Fatty Acids Carried by Lipoproteins: Revisiting the Overlooked Cargo

**DOI:** 10.1111/apha.70296

**Published:** 2026-08-21

**Authors:** Gregory C. Henderson, Kimberly K. Buhman, Keigo Tomoo

**Affiliations:** ^1^ Department of Nutrition Science Purdue University West Lafayette Indiana USA

**Keywords:** fat metabolism, FFA carrier proteins, FFA transportome, NEFA, nonesterified fatty acid

## Abstract

Free fatty acids (FFAs) are hydrophobic and therefore cannot enter the bloodstream at appreciable levels unless they are solubilized by FFA carriers. Albumin has been appreciated for years as the major FFA carrier, but it is not the only carrier. While the cholesterol and triacylglycerol components of lipoproteins are measured clinically, there are other bioactive lipids present in lipoproteins as well. There is strong evidence showing that multiple lipoprotein classes carry FFAs, and some evidence indicates potential physiological roles of lipoprotein‐bound FFAs. Apolipoproteins and other lipoprotein‐associated proteins may be responsible for FFA binding to lipoprotein particles, as lipoproteins contain numerous proteins that are predicted to have multiple FFA binding sites. Lipoproteins isolated from plasma contain FFAs, and the FFA loading appears to be driven in part by mass action when the FFA level is elevated as well as by low albumin abundance in cases of severe hypoalbuminemia. When the FFA levels rise on lipoproteins, through effects on cholesterol ester transfer protein, the lipoprotein profile may be altered. FFA binding to lipoproteins may also protect the lipoproteins from oxidative damage. The contribution of lipoproteins to FFA flux through the circulation is less clear but may play a particularly important role when albumin expression is low to drive the recruitment of this secondary FFA carrier system. In recent decades research progress has slowed in this area, leaving major knowledge gaps to address. The remaining need for research is discussed, and the physiological implications of lipoprotein‐bound FFAs are discussed in this review.

## Practitioner Points

- There is strong evidence that free fatty acids can be carried by lipoproteins.
- Rising plasma free fatty acid concentrations may promote binding of FFAs to lipoproteins.
- Free fatty acid trafficking by lipoproteins may play a particularly important role in the body when albumin expression is low.

## 1 Background

The majority of lipid storage in the body is in the form of triacylglycerol (TAG) in adipose tissue. Free fatty acids (FFAs), also referred to as nonesterified fatty acids (NEFAs), are released by lipolysis at accelerated rates when the body's need for lipids as fuel is increased, such as during fasting, exercise, hypoglycemia, and other forms of physiological stress [1]. FFAs are hydrophobic and therefore cannot enter the aqueous bloodstream at appreciable levels unless they are solubilized by FFA carriers in blood. The majority of the circulating FFA pool (primarily composed of long chain FFAs) is typically carried by albumin, the most abundant protein in blood with up to seven FFA binding sites [1, 2, 3]. The physiology of FFA transport in the circulation is a centrally important factor in human health, as higher plasma FFA concentrations under resting conditions are associated with the incidence of numerous health conditions, including insulin resistance, metabolic dysfunction‐associated steatotic liver disease (MASLD), and type 2 diabetes [4]. In agreement with a role of FFA carriers in determining the FFA level in the circulation and the organism's health, we have shown that albumin knockout mice (completely lacking serum albumin) exhibit reduced plasma FFA concentration and improved markers of insulin sensitivity [5, 6, 7].

Often albumin is considered to be the only carrier of FFAs in blood [8, 9, 10, 11]. However, it is not the only plasma protein to carry FFAs. For example, the plasma proteins vitamin D binding protein [12], retinol binding protein [13, 14], α‐1‐antitrypsin [15], and α‐2‐HS‐glycoprotein (fetuin‐A) [16, 17] can bind to FFAs. With the realization that there could be numerous proteins carrying FFAs in the bloodstream, we carried out studies of mouse plasma employing a fatty acid pulldown assay, in which proteins are isolated that bind to FFA‐conjugated agarose beads. The FFA species used were palmitate or oleate, each long chain FFAs. In this work we discovered that a very broad range of proteins may carry FFAs, including the expected proteins albumin, vitamin D binding protein, retinol binding protein, α‐1‐antitrypsin, and fetuin‐A [18, 19]. Amongst the > 200 proteins that we discovered, those exhibiting higher protein abundance included numerous apolipoproteins, including apolipoprotein A‐I (Apo AI) and apolipoprotein B‐100 (Apo B100), the major apolipoproteins of high density lipoprotein (HDL) and both very low density lipoprotein (VLDL) and low density lipoprotein (LDL), respectively. Further, when we carried out fast protein liquid chromatography (FPLC) to fractionate lipoprotein particles from mouse plasma, FFAs co‐eluted with lipoproteins, particularly with the HDL fraction which is the most abundant lipoprotein class in mice [19]. This observation of FFA transport at first glance would appear incongruent with known functions of lipoproteins, namely transport of fatty acids esterified to glycerol as TAG and to cholesterol as cholesterol ester (CE). However, as discussed below, the lipidome of lipoproteins contains various other lipids as well, and in that light it becomes less surprising that they may also carry FFAs.

In lipidomics studies (when studying HDL [20, 21, 22, 23, 24, 25, 26, 27, 28, 29], LDL [24, 25, 26, 27, 28, 29, 30, 31], VLDL [24, 25, 26, 27, 28, 29, 32], or chylomicrons [24, 25, 33, 34, 35]) and in clinical analyses [36], it is not customary to measure FFAs in lipoproteins. In a few limited exceptions, FFA data were reported in lipidome results for lipoproteins [37, 38]. One may wonder if all important cargo of lipoproteins is widely appreciated these days. Despite it being generally uncommon to study FFAs in lipoproteins, if one looks back at the literature on lipoprotein biology back to the mid‐20th century, it then becomes clear that there is abundant evidence that lipoproteins carry FFAs. That evidence and its physiological implications are reviewed below. Lipoproteins are most often described as carrying phospholipids at the perimeter with CE and TAG in their core, without consideration of other lipids. However, the diversity of lipids in lipoproteins is very complex, including lipids such as lyso‐phospholipids, diacylglycerol, sphingolipids, and free cholesterol [21, 22, 23, 24, 26, 27, 28, 30, 31, 32]. Further, of focus in the present manuscript, lipoproteins appear to contain FFAs.

As discussed in this review, there is strong evidence that lipoproteins can carry FFAs. The focus of this review is long chain FFAs, the most abundant FFA type in the circulation. To our knowledge, it is unknown if lipoproteins carry short chain fatty acids, and the information reviewed about plasma FFAs here is expected to be particularly relevant to the transport of long chain FFAs. Much of the data supporting lipoprotein‐mediated FFA transport in blood were reported many decades ago with greatly diminished contributions on the topic in the past two decades, despite the exciting promise of this research area. Important progress was made, but slowed efforts on the topic left major knowledge gaps. Increased attention to the FFA cargo in lipoproteins might lead to discoveries that would extend our understanding of physiology and the regulation of health, and therefore the hope is that this review could act as a catalyst to encourage our scientific community to more often contemplate this potentially important cargo molecule when studying lipid metabolism.

## 2 Indirect Evidence That Lipoproteins Carry FFAs

Some initial indirect evidence for lipoproteins carrying FFAs was reported in the 1950s, in which investigators noticed that the electrophoretic mobility of lipoproteins increased when they were incubated with an FFA (oleate). The authors reasoned that the oleate bound to lipoproteins, imparting negative charges, increasing the particles' movement in an electric field and suggesting a physical relationship between FFAs and lipoproteins [39]. Since this time, additional modes of evidence have emerged, such as from proteomics studies.

Lipoproteins contain proteins at their surface which are expected to have an inherent capacity to carry FFAs. We recently discovered that a wide range of plasma proteins may be capable of carrying FFAs. By employing a fatty acid pulldown assay, over 200 putative FFA carrier proteins were identified by our team [18, 19]. A subset of these proteins (the most abundant 23 proteins) in the dataset were then investigated by docking simulations to predict whether they contained FFA binding sites deemed to be moderate‐ or high‐affinity sites [19]. In this work for estimating predicted binding affinity of the FFAs palmitate and oleate to plasma proteins of interest, the affinity was estimated by a scoring function that predicts the change in free energy when a ligand binds to a protein, based on the predicted 3D structure of each protein and of each FFA. Docking results verified the likelihood that the fatty acid pulldown assay was identifying FFA carrier proteins, as all 23 proteins were predicted to contain at least one FFA binding site, with 20 of the proteins containing at least one high affinity site and 3 of the proteins containing what we deemed to be moderate affinity binding sites. When initially analyzing the putative FFA carrier protein data, we noted that some of these proteins are classically considered to be lipoprotein bound (i.e., numerous apolipoproteins) [18, 19]. Further, docking confirmed that Apo AI (the major surface protein of HDL) is predicted to have more than 10 FFA binding sites. Docking also indicated that Apo B100 (the major surface protein of LDL and VLDL) is also predicted to have numerous (~100) FFA binding sites. In addition to HDL, LDL, and VLDL containing apolipoproteins that might carry FFAs, it is noted that the identification of Apo B100 by proteomics may have also been a result of detecting peptides from Apo B48, a truncated form of Apo B100 which is on chylomicrons in humans and on chylomicrons, VLDL, and LDL in mice.

The evidence for FFA carrier proteins at the surface of lipoproteins also goes beyond Apo AI and Apo B100. As discussed below, the majority of the proteins known to reside on lipoproteins have also been discovered to be putative FFA carrier proteins by our research group [18, 19]. It is possible that each of these many proteins has a strong affinity for FFAs, or it could be that each lipoprotein class has a major FFA carrier at the surface (e.g., Apo AI or Apo B100) which is pulled down by the fatty acid pulldown assay, bringing the other components of the lipoprotein with it, making it appear that lipoproteins contain more FFA carriers than they really do. This is unknown but points to the importance of utilizing multiple lines of evidence when considering whether a protein may be an FFA carrier, such as docking or biochemical experiments in addition to pulldown assays on plasma. As described above, we also used docking simulations to identify potential carriers of FFAs as an additional form of evidence. Below, the proteomes of HDL, LDL, VLDL, and chylomicrons are discussed, and it is noted which of these proteins have been verified by docking to be potential FFA carriers.

A diverse array of proteins have been detected by proteomic analysis of isolated HDL from humans [40, 41] and mice [42, 43]. When our team performed a fatty acid pulldown assay on unfractionated mouse plasma, the majority of the proteins from each of these HDL proteome papers were identified in our fatty acid pulldown assay [19]. Further, many of the HDL‐associated proteins identified in mouse plasma and human HDL have been shown by us using docking to be likely FFA carrier proteins [19], including Apo AI, Apo AIV, transferrin, and > 10 others. It is also noteworthy that studying purified Apo AI and transferrin in vitro, we have further confirmed each of their propensity to bind to FFAs [19], indicating further support for the docking data.

Based on proteomics analysis, a broad range of proteins have also been detected in LDL from humans [44, 45] and pigs [46] as well as through analysis of an ApoB‐containing lipoprotein fraction from mice that contained both LDL and VLDL [43]. As noted above for HDL, using a fatty acid pulldown assay [19] we observed that the majority of proteins discovered in isolated LDL by proteomics are further characterized as putative FFA carriers by this orthogonal method, with multiple proteins in each paper [43, 44, 45, 46], around 10 in total including Apo B100 [45] shown by us using docking to be predicted to contain FFA binding sites [19]. Multiple proteins have been detected by proteomic analysis of human VLDL [45, 47], the majority of which were identified by us as putative FFA carriers [19], with multiple proteins in each paper predicted by docking to contain FFA binding sites, including Apo B100 [19].

While less appears to be known about the global proteome of chylomicrons than that of other lipoprotein fractions, some information is known about proteins which circulate bound to chylomicrons. From targeted assays in non‐omics studies, chylomicrons are known to contain Apo B48, Apo AI, Apo AII, Apo AIV, Apo AV, Apo E, Apo CI, Apo CII, and Apo CIII [48, 49, 50]. The majority of the proteins known to be carried by chylomicrons have also been identified by us as putative FFA carriers by a fatty acid pulldown assay [19], with Apo AI and Apo AIV predicted to contain FFA binding sites [19]. Further, Apo B48 might have multiple binding sites for FFAs, as we have shown that Apo B100 is predicted to have ~100 moderate‐to‐high affinity FFA binding sites, and the amino acid sequence of Apo B48 is similar to the 48% of the protein at the N‐terminus side of Apo B100. Proteomics analysis of chylomicrons to our knowledge has not been reported, aside from a study on remnants. In the report on proteomics analysis of isolated chylomicron remnants [51], the majority of the discovered proteins have also been identified as putative FFA carriers by a fatty acid pulldown assay, with multiple proteins predicted to contain FFA binding sites based on docking results [19]. It appears that the full range of major lipoprotein classes may contain FFA carrier proteins, providing support and a potential mechanism for the idea that they could carry FFAs.

## 3 Direct Evidence That Lipoproteins Carry FFAs

Typically in clinical analyses of plasma lipids, total cholesterol (sum of CE and free cholesterol) and TAG are measured in plasma, and cholesterol (again as a sum of CE and free cholesterol) is measured in HDL and either calculated or directly measured in LDL [36]. Total plasma FFA concentration is often measured in research; though it can be measured clinically as well, it is not included in the traditional lipid panel for assessing risk of cardiovascular disease. In contrast to analyses of cholesterol, in the clinical setting FFA concentration is not measured in lipoproteins. However, in the research setting, important discoveries have been made through the analysis of FFAs in lipoproteins. Well beyond a half century ago, it was discovered that labeled FFA tracers will partition into lipoprotein fractions of human serum in vitro, based on the use of ^14^C‐labeled long chain FFAs (palmitate, oleate, stearate, and linoleate) as well as a ^14^C‐labeled form of the medium chain FFA laurate [52]. However, additional steps along the translational spectrum would be needed to justify routine analysis of this lipid in the clinical space. Further support for the idea of FFAs in lipoproteins could come from the many lipidomics studies that are regularly published, if indeed FFAs were to be measured in these methods. However, esterified lipids are typically the focus in lipidomics, with FFAs usually not monitored by the methods. In lipidomic analyses of isolated HDL particles from human plasma, typically FFAs were apparently not monitored by the method [20, 21, 22, 23, 24, 25, 26, 27, 28, 29], and therefore it was unknown if FFAs were HDL cargo molecules. In lipidomic analyses of isolated LDL [24, 25, 26, 27, 28, 29, 30, 31] or VLDL [24, 25, 26, 27, 28, 29, 32], typically FFAs were also apparently not monitored by the method. However, we are aware of a single publication reporting from lipidomic analysis that LDL contains FFAs [38], as a component of the very broad spectrum of different lipid classes carried by LDL. Overall, because of the limited data on FFAs from lipidomics research in the past, the literature on non‐omics studies is crucial for determining the potential extent to which HDL, LDL, and VLDL might carry FFAs.

**FIGURE 1:**
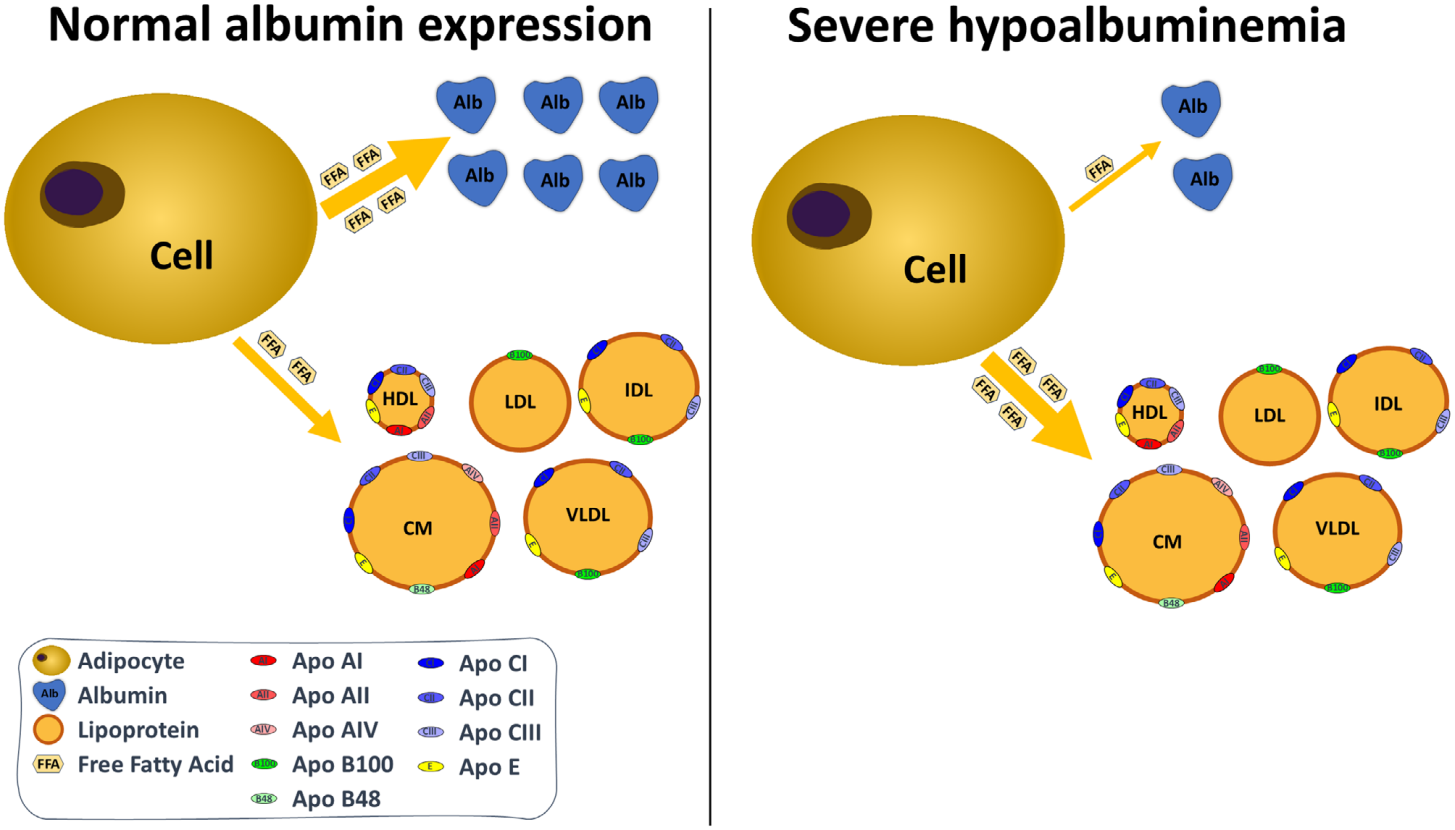
FFAs as cargo for albumin and lipoproteins in health and in severe hypoalbuminemia. The FFA carrying capacity (maximum of 7 FFAs per albumin) is underutilized in normal physiology but is nearly maximized when albumin expression is low. When FFA carrying by albumin is nearly saturated, or when albumin expression is completely absent, the relative proportion of FFAs on lipoproteins rises. The rank order of lipoprotein particle size (CM > VLDL > IDL > LDL > HDL) is shown, but precise proportionality is not shown to scale. There is strong evidence that each of these lipoprotein classes carry FFAs in the bloodstream, but it is not yet clear if a certain class is significantly more involved in this FFA trafficking process than the others. CM, chylomicron; FFA, free fatty acid; HDL, high density lipoprotein; LDL, low density lipoprotein; VLDL, very low density lipoprotein.

HDL, VLDL, and LDL, isolated by ultracentrifugation, were shown to carry FFAs in plasma of hypertriglyceridemic humans under basal conditions and then could carry extremely high FFA levels after stimulating in vitro lipolysis with LPL [53]. The in vitro study was useful to show the reserve capacity in lipoproteins for carrying FFAs, but it should also be noted that the conditions did not represent the normal physiology of a healthy individual. In response to treatment with LPL in vitro, FFAs were elevated beyond albumin's carrying capacity, such that albumin's FFA binding would be likely saturated, forcing lipoproteins to carry FFAs. Indeed, this may be one of the functions of lipoproteins, to carry FFAs if albumin expression or its FFA carrying capacity is insufficient (Figure 1).

Nephrotic syndrome, a subset of chronic kidney disease in which patients experience severe hypoalbuminemia, can be studied as a natural example of lipid trafficking in the presence of low serum albumin levels. FFA levels in HDL, VLDL, IDL, and LDL, each isolated by ultracentrifugation, tended to be enhanced in nephrotic syndrome patients, with the FFA elevation in LDL reaching significance [54]. This study was in agreement with an early paper from the 1950's (before the use of modern lipoprotein terminology and lipid analysis methods), based on densities of ultracentrifugation fractions showed nephrotic syndrome patients had higher FFA levels in a fraction containing both IDL and VLDL as well as in the LDL fraction [55]. Further support for the effects of nephrotic syndrome comes from a rat study. In a rat model of nephrotic syndrome, FFA transport was challenged by injection of heparin into the rats to stimulate the generation of FFAs in the bloodstream by promoting lipolysis of the TAG in lipoproteins [56]. The authors showed that under basal conditions HDL, VLDL, IDL, and LDL (isolated by ultracentrifugation) circulate with FFAs bound to them. Even after heparin injection, still the majority of the plasma FFA pool was associated with the free protein fraction (presumably albumin mostly) in healthy control rats, while in rats with nephrotic syndrome after heparin injection most FFAs were bound to lipoproteins (especially to HDL) [56]. In patients with severe hypoalbuminemia without kidney disease, as well as in control subjects, lipoproteins that were isolated by ultracentrifugation (HDL, VLDL, IDL, and LDL) were again shown to contain FFAs, and the level of FFAs bound to HDL and LDL was greater in the hypoalbuminemia patients than in healthy subjects [57]. Finally, additional evidence for the body's ability to recruit HDL and LDL for carrying FFAs comes from a study of extreme exercise in humans [58]; in the fasted state, men ran for 4 h, raising the plasma FFA level greater than 4‐fold over resting levels. After exercise, FFAs were elevated by approximately 5‐fold in LDL and about 10‐fold in HDL, based on analyses of lipoprotein fractions isolated by ultracentrifugation. However, the authors did report some contamination of albumin in HDL, so it is possible that FFA content in that fraction was overestimated. When we performed western blot for albumin in HDL fractions from FPLC of mouse plasma, we did not detect any albumin, yet still we detected FFAs in these fractions [19]; thus, while it is possible that FFA levels in HDL could have been overestimated when using ultracentrifugation, we do believe the discovery of FFAs in HDL is still generally valid and not entirely the result of albumin contamination, although the quantitative results should be interpreted with caution.

Beyond the studies discussed above, further evidence about lipoprotein‐mediated FFA trafficking through the bloodstream comes from studies specifically focusing on LDL analysis. It has been shown in these studies that LDL in human plasma is bound to FFAs through discovery that FFAs are contained in the LDL fraction isolated by ultracentrifugation alone [59] and by ultracentrifugation followed by anion exchange chromatography [60, 61]. Further evidence for FFA binding to lipoproteins comes from the subset of LDL that takes on a more negative charge than the majority of LDL. This electronegative LDL has elevated levels of FFAs, as compared with normal (“electropositive”) LDL, and electronegative LDL levels are higher in type 1 and type 2 diabetes [60, 61] as well as in dyslipidemia and heart disease [62].

Chylomicrons are TAG‐rich lipoproteins synthesized by the small intestine that carry dietary TAG and cholesterol through the lymph and then through the bloodstream. In the only published lipidomics analyses of isolated chylomicrons [25, 33, 34, 35] or chylomicron remnants [24] from human plasma, FFAs were not measured. However, in one study of chylomicrons from rat lymph, FFAs were analyzed by lipidomics and the authors reported that chylomicrons from rat lymph contained FFAs [37] alongside a very broad spectrum of various other lipid classes. This is in agreement from targeted measurements of lipids in chylomicrons from dog lymph, indicating that chylomicrons contain low levels of FFAs, potentially both in the phospholipid monolayer and in the core [63]. Unlike the case for HDL, VLDL, IDL, and LDL discussed above, we are not aware of any literature testing the physiological implications of chylomicron‐bound FFAs, and generally the body of literature is far more extensive for these other lipoprotein classes in comparison to chylomicrons. Although chylomicron research is at an earlier stage of investigation with regard to the function of FFAs in this lipid carrier, when considering all lipoprotein classes collectively, we conclude there is overall strong evidence supporting the notion of FFA trafficking by lipoproteins.

## 4 Regulation of FFA Binding to Lipoproteins

**FIGURE 2:**
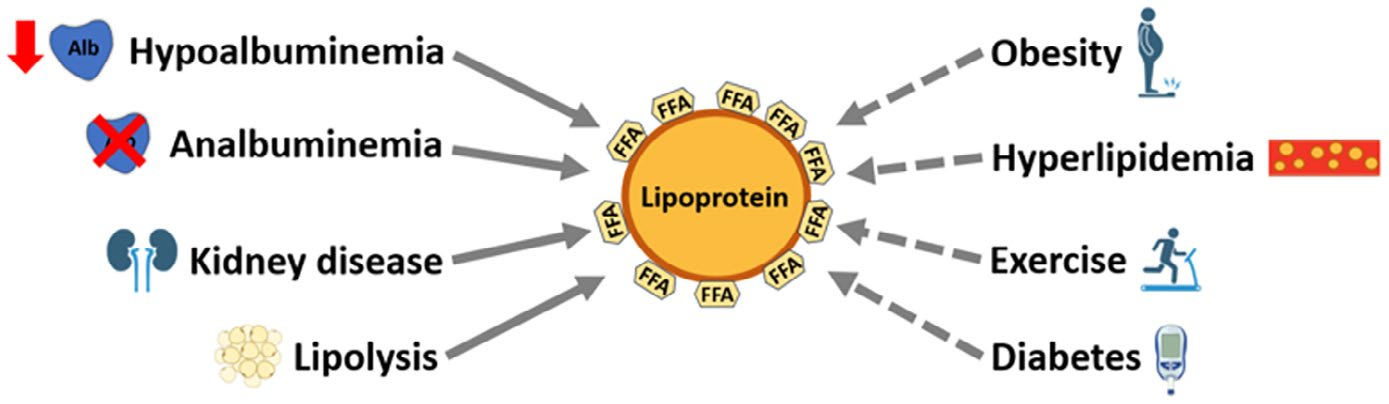
Schematic describing the factors that promote FFA binding to lipoproteins. The factors to the left are supported by strong evidence, while the factors to the right have some limited evidence. Kidney disease refers particularly to nephrotic syndrome in which serum albumin levels are very low. Lipolysis potentially refers to both that occurring in adipose tissue and in the vascular space. The evidence for diabetes promoting FFA binding to lipoproteins is stronger for type 2 diabetes than for type 1 diabetes.

**FIGURE 3:**
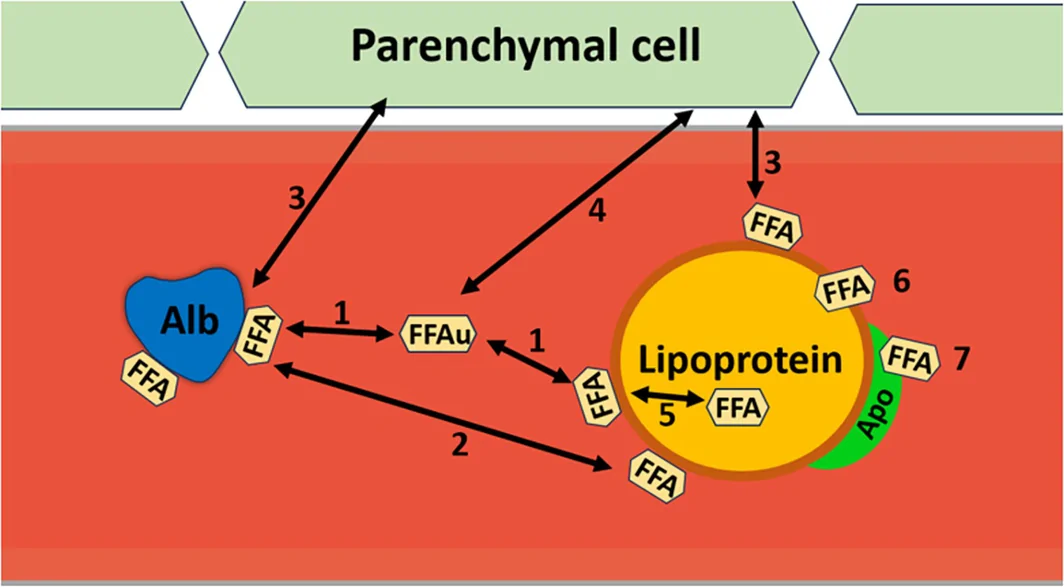
Potential roles of FFAs that are albumin‐bound, lipoprotein‐bound, and unbound (FFAu) in cellular uptake and release of FFAs from the bloodstream. FFAs carried by albumin and lipoproteins may dissociate into the form of FFAu before uptake into cells (1). It is also possible that FFAs directly transfer between albumin and lipoproteins without entering an unbound state (2); this would require close proximity of albumin and lipoproteins at the time of transfer. When FFAs enter and exit the bloodstream, they might be passed directly to carriers without entering the unbound state (3) or they might enter the unbound state briefly upon entry into the bloodstream (4). While it is assumed FFAs resided at the surface of lipoproteins, it is also possible that some FFAs reside inside the lipid core of lipoproteins along with neutral lipids such as triacylglycerol and cholesterol ester and then are transferred outward for release (5). Additionally, it could be that instead of sitting at the surface of lipoproteins bound to the edge of the lipoprotein particle, the FFAs might penetrate into the phospholipid monolayer (6) or they may be bound directly to apolipoproteins (7). Alb, albumin; Apo, apolipoprotein; FFA, free fatty acid; FFAu, unbound free fatty acid. See Figure 1 for definition of the objects within the figure image.

There are various potential determinants of the FFA loading on lipoproteins (Figure 2). The circulating concentration of plasma FFAs might be one determinant of the FFA abundance which will bind to lipoproteins. Injecting heparin into hypertriglyceridemic patients raised the total FFA concentration to high levels (~1.4 mM) and caused a small increase in FFA on lipoproteins, but the vast majority of FFAs remained on albumin [53]. In another study, heparin injection in humans was not sufficient to raise the FFA level on LDL [59]. However, in the same study, in vitro lipolysis of plasma caused an increase in FFAs on LDL [59], and so it appears that the threshold for raising FFAs to load lipoproteins may be high, although an extreme bout of long‐duration exercise in fasted humans led to a substantial rise in the total plasma FFA concentration as well as the levels bound to LDL and HDL [58]. Loading of FFAs onto lipoproteins may also be regulated by the availability of other FFA carriers. For example, we have observed that in albumin knockout mice the binding of FFAs to HDL as compared with binding to free proteins was proportionally increased in albumin knockout mice compared to wildtype controls [19]. When LDL was isolated from human plasma and then incubated with albumin, adding albumin decreased the amount of FFA on HDL, VLDL, and LDL [53, 64], indicating that in vitro albumin availability can negatively regulate FFA binding to lipoproteins. In agreement, nephrotic syndrome patients (who have low serum albumin levels) have over a third of their plasma FFAs carried by lipoproteins in comparison to < 10% in control subjects [54]. Similarly, in a rat model of nephrotic syndrome, after heparin injection to raise plasma FFA concentration, most FFAs are bound to lipoproteins, especially to HDL, whereas in healthy control rats the majority of FFAs remain bound to free proteins [56]. Thus, it appears that the plasma FFA level as well as albumin abundance are determinants of the lipoprotein‐bound FFA level. When the capacity for albumin approaches being full, then lipoproteins become more important. However, the extent to which albumin's binding sites must be occupied to promote lipoprotein‐FFA binding remains unclear (Figure 3). Other factors that might also impact lipoproteins carrying FFAs are the circulating LDL level when elevated by obesity [65], as well as limited evidence that type 2 diabetes might raise FFAs in LDL [60].

## 5 How Much FFAs Bind to Lipoproteins

Our research group has analyzed plasma from wildtype and albumin knockout mice on numerous occasions, with assays of FFAs, TAG, and total cholesterol in each fraction from FPLC. When quantitating the amount of FFAs in lipoproteins, there is no consensus on how to normalize the data. From our data on FFAs in lipoprotein fractions of mouse plasma, in which total cholesterol and FFAs were measured in isolated lipoproteins, we have an opportunity to normalize FFAs to the cholesterol level. From our results [19, 65], it appears that typically the molar ratio of FFAs to total cholesterol in HDL is approximately 0.1 and in the VLDL/LDL fraction is around 0.1–0.2. In another study, in human plasma the FFA/cholesterol ratio was around 0.1 (VLDL: 0.06; LDL: 0.07, HDL: 0.13) [53]. Thus, it appears that FFAs on lipoproteins are approximately 10% of the cholesterol level in these lipoproteins, or sometimes higher. Under in vitro lipolysis conditions that drastically raise the FFA abundance in solution, far more FFAs can bind to lipoproteins; in this case the lipoproteins can contain more FFA than cholesterol (FFA/cholesterol ratios higher than 1 in vitro for VLDL, LDL, and HDL) [53]. Clearly, cholesterol is the main cargo of these lipoproteins, but there does appear to be a meaningful level of FFAs as well in the lipoproteins. Another approach for normalizing FFA levels in lipoproteins (for ApoB‐containing lipoproteins) would be to take the molar ratio of FFAs to ApoB. In humans, the FFA/ApoB ratio in LDL at rest was approximately 4 and after an extreme bout of fasted exercise was approximately 19 [58]. This capacity to reach a high ratio is in agreement with our group's prediction of an immense number of binding sites on Apo B100 for FFAs using in silico modeling [19]. It was also reported that in human LDL the molar ratio of FFA/ApoB is 13–15 in electropositive LDL and 27–40 in electronegative LDL [61]. The FFA/ApoB ratio in normal (electropositive) LDL was shown in another study to be ~16 in healthy subjects, ~24 in well‐controlled T2D, and ~28 in poorly controlled T2D. The ratio in electronegative LDL was ~47 for controls, ~60 for well controlled T2D, and ~76 for poorly controlled T2D [60]. We are not aware of published data describing the quantitative level of FFAs in other lipoprotein types aside from HDL, LDL, and VLDL, such as chylomicrons.

## 6 Potential Importance of Lipoprotein‐Bound FFAs in Physiology and Regulation of Health

There appear to be multiple manners in which lipoprotein‐bound FFAs could impact physiology and health. One impact is through the effects on the activity of cholesterol ester transfer protein (CETP), an enzyme that transfers CE from HDL to ApoB‐containing lipoproteins (VLDL and LDL), while simultaneously transferring TAG from ApoB‐containing lipoproteins to HDL. One of the most noteworthy actions of CETP is to decrease the circulating level of HDL cholesterol. When the FFA level bound to LDL and HDL rises, this increases the electronegativity of the lipoprotein particles [54, 56, 57, 58, 62], and as a result of this electronegativity the activity of CETP rises [54, 57]. As noted above, patients with nephrotic syndrome have severe hypoalbuminemia and ultimately carry more FFAs on lipoproteins than in healthy individuals, likely because of the reduced abundance of albumin. Patients with nephrotic syndrome exhibit elevated electronegativity of both HDL and LDL [54]. Not only do they have higher CETP expression, but also because of the electronegativity they have increased specific activity of CETP in the circulation. These patients have low HDL cholesterol (HDL‐C) and high LDL cholesterol (LDL‐C), as well as increased TAG in HDL and decreased TAG in VLDL and LDL (consistent with actions of CETP). In patients with severe hypoalbuminemia, likely resulting from a heritable mutation in the albumin gene, there is also elevated LDL electronegativity and high CETP activity in plasma, although HDL electronegativity was not apparently studied [57]. Furthermore, adding albumin in vitro to the plasma from these hypoalbuminemia patients reduced the CETP activity [57], presumably because albumin pulled FFAs away from lipoproteins, thus reducing the charge on the particles. Aside from the patient populations mentioned above, other groups with elevated LDL electronegativity include type 2 diabetes, especially those with poorly controlled type 2 diabetes, and indeed their electronegative LDL contains higher levels of FFAs than their electropositive (normal) LDL [60]. With regard to cardiovascular health, FFAs on lipoproteins may have effects that go beyond the modulation of CETP as well. In another study, HDL was loaded with FFAs in vitro, generating FFA‐loaded HDL with an electronegative charge, and then this HDL was injected into rabbits. It was seen that the fractional catabolic rate of HDL declined when it was loaded with FFAs. Ultimately in this work it was seen that FFAs on HDL lead to a slowed HDL clearance from circulation, or specifically slowed breakdown of Apo A1 [66]. The physiological impacts of this altered HDL kinetics are not yet known but the finding indicates another potentially important impact of FFAs on lipoproteins.

While one might presume that lipoprotein electronegativity and CETP activation would have negative health impacts, it is too soon to vilify the FFA cargo on lipoproteins. It appears that there may also be beneficial impacts of this cargo molecule. Oxidized LDL is atherogenic and generally considered to be physiologically harmful. Incubating LDL with high levels of FFAs, leading to increased FFAs on the lipoprotein particles, leads to protection from oxidation in vitro [58, 64, 67]. Further, when LDL isolated from albumin‐deficient patients (analbuminemic individuals) was incubated with albumin, this treatment decreased the amount of FFA on LDL, and LDL became more prone to oxidation when it lost its FFAs in this case. So, it appears that FFAs on lipoproteins may serve a protective function in addition to potential detriments, while the other main carrier of FFAs (albumin) has negative impacts on LDL oxidation.

Potentially because of a weaker binding of FFAs to lipoproteins than albumin, it was shown that incubating mouse macrophages with FFAs bound to VLDL, LDL, or HDL killed the cells, but FFAs bound to albumin caused much less toxicity [53]. In this paper it was also shown that LDL‐bound FFAs caused more TAG accumulation than albumin‐bound FFAs in cultured macrophages [53]. It has also been shown that FFA‐loaded LDL causes inflammation in cultured endothelial cells, although in this study there was no comparison to albumin‐bound FFAs [67]. The findings of inflammation should not be surprising, as we know that FFAs carried by other FFA carriers such as albumin would also cause this effect, as FFAs per se promote inflammation [68, 69]. Nonetheless, the findings are noteworthy in that they show LDL can carry FFAs which will exert influence on cellular function [67]. In another study that did not compare to albumin‐bound FFAs, it was discovered that FFA‐loaded LDL increases TAG, FFA, and reactive oxygen species levels in cardiomyocytes [70]. The lipotoxicity in vitro of lipoprotein‐bound FFAs is interesting but should also be considered in the context of the full plasma/blood matrix of a live animal which would also contain high amounts of albumin (with the exception of congenital analbuminemia patients and albumin knockout rodents). In vivo, albumin is always present in normal animal physiology, and thus cells are not presented with lipoprotein‐bound FFAs without albumin present, and so it is important to consider their functions in the presence of albumin. In a study of breast cancer cells (Ehrlich ascites cells), the investigators did give such consideration to this issue. The authors studied ^14^C‐labeled FFAs and determined that uptake of FFAs into cells carried by LDL, VLDL, or HDL was quite rapid. The cells stored a significant amount of the FFAs and oxidized FFAs to generate carbon dioxide [71]. They determined that the cells do not take up the entire LDL particles but simply took up the FFAs from the lipoprotein. Importantly, they determined that the uptake of FFAs from LDL was slowed substantially when albumin was added to the media. Thus, likely albumin stripped the FFAs away from LDL to a significant extent and then sequestered the FFAs in the media rather than allowing a rapid cellular uptake. Furthermore, after FFA uptake from LDL in the absence of albumin, when the investigators added new media with albumin, then much of the intracellular radioactivity (from the radiolabeled FFAs) was immediately released back to the media [71]. This further suggests that sustained FFA uptake from LDL is inhibited by albumin and shows that albumin promotes FFA release from cells. This finding about albumin promoting FFA release is in agreement with our finding that albumin knockout mice exhibit slowed FFA flux in the bloodstream [72]. Overall it appears that the effects of lipoprotein‐bound FFAs are quite pleiotropic, with some possible benefit and some possible detrimental effects, and we note here that results from in vitro experiments must be interpreted with caution when the culture media does not approximate the complexity of animal plasma.

As discussed above, it has been shown in cell culture experiments that cells can take up FFAs from lipoprotein particles [70, 71]. This suggests the possibility that delivery of FFAs to tissues could be the primary reason why lipoproteins carry FFAs. The relative importance of fuel delivery as compared with functions such as CETP modulation and regulation of oxidative damage is unknown, but at least in certain circumstances, the shuttling of FFAs from storage sites to utilization sites by lipoproteins may be very important. For example, in the instances that albumin expression is very low, lipoproteins become a relatively major carrier of FFAs. Historically, albumin has been considered by some to be the sole carrier of FFAs. While it is not the sole carrier, it is a very major FFA carrier. We have shown that in albumin knockout mice, the plasma FFA concentration [5, 6, 7] and flux rate [72] are reduced by approximately 2‐fold, indicating that a significant amount of FFAs circulate even in the absence of albumin, and these hydrophobic molecules would need a carrier to remain in solution. We have discovered numerous proteins that may carry FFAs, collectively termed the plasma FFA transportome [18, 19], and many of these proteins are known to circulate bound to lipoproteins.

Importantly, by fast protein liquid chromatography, we showed that FFAs can be carried by HDL as well as by ApoB‐containing lipoproteins [19, 65]. Indeed we showed in albumin knockout mice that particularly HDL becomes a predominant carrier of FFAs in the case of albumin deficiency, carrying more FFAs than free proteins [19]. Furthermore, in albumin knockout mice the amount of FFA flux, perhaps promoted largely by HDL, is still sufficient to maintain a normal rate of whole body fat oxidation [5, 6]. Thus, the redundancy in systems for FFA trafficking in the body allows for sufficient FFA flux even when albumin is absent. This idea of HDL promoting FFA flux is further supported by the observation that overexpression of Apo AI in mice raises the total plasma FFA concentration [73]. Further agreement with this observation comes from a study of transgenic mice overexpressing CETP. In these mice, when CETP was inhibited by a drug which led to elevated Apo AI and HDL cholesterol, the total plasma FFA concentration rose [74]. These two transgenic mouse studies [73, 74] do not prove that Apo AI carries FFAs, but they are generally supportive of our observation that HDL particles do carry FFAs [19]. In certain physiological states lipoproteins may become particularly important carriers of FFAs for promoting fuel flux around the body, and further support for this notion comes from studies of patients with nephrotic syndrome. As noted above, approximately one third of FFAs are lipoprotein‐bound in nephrotic syndrome patients [54], and over half are lipoprotein bound in patients with severe hypoalbuminemia that likely resulted from a heritable mutation in the albumin gene [57], whereas in healthy control subjects not more than 10% of plasma FFAs are typically carried by lipoproteins [54, 57]. The 10% of FFAs on lipoproteins might be physiologically meaningful, but especially when the higher percentages of circulating FFA are on lipoproteins, then this form of FFA shuttling must rise to central physiological relevance, and indeed the FFA level in LDL and HDL was significantly enhanced by hypoalbuminemia [57] potentially as a means of maintaining FFA delivery to tissues for β‐oxidation and biosynthetic processes. Thus, a major role of lipoprotein‐bound FFAs may be the shuttling of FFAs between tissues, especially when serum albumin levels are compromised.

As described above, there are many potential impacts of FFA trafficking by lipoproteins, and those with evidence from the literature are discussed above. One could also speculate about other reasons why FFAs are carried by lipoproteins. In addition to the FFA binding to albumin that would occur, FFAs might be temporarily stored inside or at the surface of lipoproteins following the lipolysis of TAG in TAG‐rich lipoproteins by LPL and hepatic lipase (HL). When there is a rapid release of FFAs from VLDL and chylomicrons by vascular lipase molecules, the high local concentration of FFAs might be handled in part by binding of FFAs to the lipid or protein components at the surface of lipoproteins until either the FFAs are transferred to albumin, transferred to other FFA carrier proteins, or taken up by tissues. When LPL acts on the TAG in chylomicrons or VLDL, most of the liberated FFAs are taken up by nearby tissues (e.g., muscle or adipose tissue) for oxidation or re‐esterified into TAG. However, spillover occurs when some of these liberated FFAs are not immediately taken up and instead enter the plasma as FFAs [75], and following spillover the FFAs may be carried away from the site of intravascular lipolysis by FFA carrier proteins (e.g., albumin) as well as potentially by lipoproteins. This spillover process is significant particularly in the postprandial state following a high fat meal, and it tends to be higher in individuals with lower muscle mass and/or with higher adiposity [76]. While FFAs are likely carried at the surface of lipoproteins, it is also possible that some FFAs are in the core of lipoproteins (e.g., intermingled with CE and TAG (Figure 3)). This CE and TAG in the core of the lipoproteins would have been primarily incorporated into the lipoprotein particle in the liver before plasma FFAs bound to the secreted lipoprotein in the bloodstream. However, it could also be that hepatocytes package FFAs into VLDL while adding the CE and TAG, thus allowing the liver a route for releasing intracellular FFAs. Finally, it is possible that lipoprotein‐bound FFAs are targeted to specific tissue types for cellular uptake, as some tissues might prefer to take up FFAs from specific FFA carriers; this is speculative, but it is noteworthy that albumin knockout mice exhibit reduced lipid in the liver more than in skeletal muscle or heart [5, 6], suggesting a potential preference in the liver for FFA uptake from albumin and potential preference in muscle for FFA uptake from other carriers such as lipoproteins. In summary, lipoproteins carry FFAs and this cargo may have important physiological implications.

## 7 Methodological Considerations

Above, numerous publications reporting the discovery of FFAs in isolated lipoproteins are discussed. One possible confounding factor in these studies could be the degradation of lipids within lipoproteins during sample collection, processing, and storage. If esterified lipids (TAG, CE, DAG, phospholipids, etc.) are spontaneously degraded, FFAs would be released, and this could lead to the apparent discovery that lipoproteins carry FFAs even if in vivo they did not. While this is a potential concern, numerous studies are discussed above in which increasing the FFA abundance around lipoproteins increased their loading with FFAs, suggesting that indeed FFAs from solution can dock onto a lipoprotein particle. Further, in vitro lipolysis of TAG (or DAG, etc.) following lipoprotein isolation would release both FFAs and glycerol, yet in FPLC fractions containing lipoproteins we did not see any detectable level of glycerol [19]. Furthermore, when we incubated lipoproteins with a fluorescent fatty acid analog, we then discovered fluorescence in the lipoprotein fractions, a finding that could not be explained by in vitro lipolysis [19]. Therefore, in vitro lipolysis alone could not explain the discovery of associations between lipoproteins and FFAs.

Another concern in studies that use ultracentrifugation to isolate lipoprotein fractions could be contamination of HDL with serum albumin. This is believed to occur in ultracentrifugation because of an overlapping density range for albumin and HDL [41, 77]. This could be a significant concern as ultracentrifugation is the most commonly employed technique for isolated lipoproteins, and contamination with albumin would bring albumin‐bound FFAs to the HDL fraction. While one should be cautious when interpreting the findings summarized above from studies that used ultracentrifugation to isolate HDL, it is very likely that this lipoprotein class does carry FFAs, as by FPLC our group has seen that FFAs circulate bound to mouse HDL, including in albumin knockout mice which express no albumin and therefore cannot have albumin contamination in their HDL fraction [19]. This FPLC‐based discovery could build confidence in the prior findings discussed above, which suggest from ultracentrifugation‐based results that human HDL contains FFAs.

Next, when measuring FFAs, one can simply measure the total FFA level or can measure each individual FFA species. The vast majority of studies discussed in this review simply measured total FFA in isolated lipoprotein fractions with an enzyme‐linked assay kit. However, it may become of interest in the future to profile different FFA species which could be carried out by gas chromatography with flame ionization detection [78, 79], high performance liquid chromatography (HPLC) with UV–Vis detection [80, 81], or by gas chromatography (GC) or HPLC coupled to a mass spectrometer (i.e., GC–MS or LC–MS) [5, 6]. When we performed a fatty acid pulldown assay using different FFA species bound to the beads (palmitic acid, oleic acid, linoleic acid, and α‐linolenic acid), each were quite similar regarding the pulled down proteome, including similar ability to pull down apolipoproteins [18]. Thus, it may be that the FFA profile on lipoproteins generally matches the FFA profile in plasma. However, in another study it was reported that lipoproteins prefer to carry saturated FFAs [53], and thus it remains possible that lipoproteins are more prone to bind certain FFA classes. When an assay kit is used to measure the total FFA concentration in plasma or in lipoprotein fractions, the traditional approach is for acyl‐CoA synthetase to add a coenzyme‐A to the FFA and then acyl‐CoA oxidase generates hydrogen peroxide that is detected in the colorimetric assay. The kits can clearly detect FFAs in plasma in which much of the FFAs are bound to albumin, and in isolated lipoproteins in which the FFAs are likely at the surface and possibly also in the core of lipoproteins; it appears that the enzymes can access the carboxylate side of the FFAs in order to catalyze the reaction. It is not known which assay type is truly the most appropriate for studying FFAs as lipoprotein cargo, but the manner in which the detection method gains access to the FFAs is quite different. In contrast to the colorimetric assays, when FFAs are measured by a chromatography‐based approach (e.g., GC–MS, LC–MS, etc.), the FFAs are first removed from their carriers by an organic extraction, typically with a mixture of chloroform, methanol, and water (with FFAs partitioning into the chloroform phase), or with a mixture of heptane and sulfuric acid (with FFAs in the heptane phase [5, 78, 79]). This likely causes the lipoproteins to dissolve and the FFA carrier proteins to denature, freeing the FFAs into solution for detection. Using chromatography and assay kits in the past have led to important discoveries, but there are advantages and disadvantages to each. The ease of the assay approach using kits is certainly a benefit, especially when a study has a high number of samples, such as many FPLC fractions, while using GC‐ or HPLC‐based approaches following organic extraction may benefit from complete removal of the FFAs from the carriers before the detection.

Another important methodological consideration when studying lipoproteins as carriers of FFAs would be the animal species selection. Rodents have relatively high HDL and lower LDL levels than humans and do not express CETP. CETP may be responsive to the FFA level on lipoproteins, but this could not be studied in mice, except for in mice that transgenically express CETP. However, there are also specific opportunities to learn about lipoproteins in mice, such as through studying albumin knockout mice or mice that lack other FFA carriers.

Another methodological consideration is how to separate lipoproteins. Ultracentrifugation or FPLC can be used. In our view, both are a reasonable choice, each with their own advantages and disadvantages. In ultracentrifugation, contamination of the HDL fraction with albumin appears to be a greater concern than in FPLC [19, 41, 77]. However, ultracentrifugation typically has a greater resolving power than FPLC for separating all lipoprotein fractions; FPLC usually does not separate LDL from VLDL in mice [43, 82, 83, 84, 85, 86], and thus the VLDL/LDL fraction is studied as a single entity, whereas in humans sometimes VLDL and LDL are separated by FPLC [26, 87, 88]. Thus, in designing each study of FFA trafficking by lipoproteins, there are important methodological factors to consider.

## 8 Knowledge Gaps

Much was learned about lipoproteins carrying FFAs in the second half of the 1900s, but attention to this topic has waned significantly, far before the scientific community could elucidate the importance of this cargo. The reason for the lost attention of lipoprotein‐mediated FFA trafficking is unclear, but it appears that the early findings did not reach an optimal impact, as these days FFAs in lipoproteins are still not measured clinically, and in lipidomics analysis of lipoproteins it is exceedingly rare for investigators to measure FFAs, even though they might measure a broad range of other lower‐abundance lipids such as ceramide, DAG, lyso‐phospholipid species, etc. It is as if much of the work on FFA trafficking by lipoproteins is underappreciated or nearly forgotten. A goal of this review is to draw attention to this potentially important cargo and ultimately catalyze future work in this area of investigation. As attention to this topic waned, the early discoveries were not advanced to the point of understanding the full physiological relevance of lipoprotein‐bound FFAs, and thus major knowledge gaps remain that should be addressed in the future. The most striking knowledge gap is related to the physiological impacts of lipoprotein‐bound FFAs. There is only very limited published information about the effects of FFA‐loaded lipoproteins on FFA uptake into cells and on the health of cells. Further, this limited work has been restricted to specific cell types. It will be important to develop a far more comprehensive understanding of the effects of this lipoprotein cargo on the regulation of metabolism in different cell types, in organs, and in live animals.

Next, it is unclear if the proteome of lipoproteins modulates their FFA carrying, yet this seems likely as we have shown that the proteome of lipoproteins contains proteins predicted to have multiple binding sites for FFAs [19]. It also remains unclear if FFAs reside on the surface or inside lipoproteins. Very likely FFAs are carried at the surface but some level of FFAs maybe also in the core. While FFAs can bind to lipoproteins from the surrounding solution (e.g., from plasma in the bloodstream), it might also be that they can bind to lipoproteins through other routes, such as binding in the liver before hepatic secretion of VLDL and HDL, and they may bind immediately after liberation from lipoprotein‐TAG by the action of LPL and HL. If FFAs are transferred from albumin to lipoproteins either directly or through release of FFAs to first become unbound FFAs (FFAu) (Figure 3), then it is likely that adipose tissue lipolysis plays a role because it promotes a rising total plasma FFA concentration and a proportional elevation of FFAu.

Another important knowledge gap would be the kinetics of FFAs bound to lipoproteins. The binding affinities, for example in comparison to albumin, to our knowledge are not yet known. The half‐life of a circulating FFA molecule bound to different lipoproteins is also unknown. The half‐life of FFAs in general in the circulation is approximately 2 min, representing a rapid turnover rate [72]. It is unknown if FFAs only spend this brief amount of time bound to lipoproteins or if, unlike albumin, the residence time of FFAs on lipoproteins is very long.

Differences between clinical populations regarding lipoprotein‐mediated FFA trafficking are also not yet clear, aside from some knowledge about congenital analbuminemia [57], nephrotic syndrome [54], and type 2 diabetes [60]. Based on our research in obese mice, it is also possible that dyslipidemia would promote carrying of FFAs by ApoB‐containing lipoproteins [65], a notion also supported by one of the first papers to show lipoproteins carrying FFAs in which “hyperlipemic” human serum appeared to at least have a tendency to have more FFAs in LDL as well as in the fraction containing VLDL with IDL, but no apparent difference in the HDL fraction [55]. It is not yet clear if the circulating level of HDL‐C and LDL‐C, or the circulating levels of Apo AI and Apo B100, determine how much FFAs will circulate bound to lipoproteins. Although there is compelling evidence that lipoproteins carry FFAs, still much work remains to understand how lipoprotein‐bound FFAs relate to health and disease.

## 9 Summary and Conclusions

In summary, there is strong evidence that FFAs can be carried by lipoproteins, but the full physiological relevance is not yet clear, and the level of progress in this area has not yet reached clinical application nor has it made sufficient impact on how investigators typically study the lipid cargo of lipoproteins in the research setting. Lipoprotein‐bound FFAs are rarely an outcome variable in metabolism and health research, despite the exciting potential for this area of study. Therefore, it appears that the earlier discoveries have been nearly forgotten. In the future, it may be important for our scientific community to generate sufficient knowledge for determining if analysis of this cargo on lipoproteins someday could be informative in the clinical space as a health biomarker or as a variable to study in mechanistic pathophysiology research. Ultimately, this line of research may lead to an impact on the understanding of physiology in health and disease as well as on how clinical chemistry of serum lipids is conducted.

## Author Contributions

**Gregory C. Henderson:** conceptualization, writing – original draft, visualization, writing – review and editing. **Kimberly K. Buhman:** writing – review and editing. **Keigo Tomoo:** writing – review and editing, visualization.

## Funding

The authors have nothing to report.

## Ethics Statement

The authors have nothing to report.

## Consent

The authors have nothing to report.

## Conflicts of Interest

The authors declare no conflicts of interest.

## Data Availability

The authors have nothing to report.
